# Adverse experiences resulting in emergency medical treatment seeking following the use of magic mushrooms

**DOI:** 10.1177/02698811221084063

**Published:** 2022-04-07

**Authors:** Emma I Kopra, Jason A Ferris, Adam R Winstock, Allan H Young, James J Rucker

**Affiliations:** 1 Department of Psychological Medicine, Institute of Psychiatry, Psychology & Neuroscience, King’s College London, London, UK; 2 Centre for Health Services Research, Faculty of Medicine, The University of Queensland, Brisbane, QLD, Australia; 3 Institute of Epidemiology and Health Care, University College London, London, UK; 4 Global Drug Survey, London, UK; 5 South London and Maudsley NHS Foundation Trust, London, UK

**Keywords:** Adverse effects, magic mushrooms, psilocybin, psychedelics, safety

## Abstract

**Background:**

Psilocybin-containing mushrooms are used for recreational, spiritual, self-development and therapeutic purposes. However, physiologically relatively nontoxic, adverse reactions are occasionally reported.

**Aims:**

This study investigated the 12-month prevalence and nature of magic mushroom-related adverse reactions resulting in emergency medical treatment seeking in a global sample of people reporting magic mushroom use.

**Methods:**

We use data from the 2017 Global Drug Survey – a large anonymous online survey on patterns of drug use conducted between November 2016 and January 2017.

**Results:**

Out of 9233 past year magic mushroom users, 19 (0.2%) reported having sought emergency medical treatment, with a per-event risk estimate of 0.06%. Young age was the only predictor associated with higher risk of emergency medical presentations. The most common symptoms were psychological, namely anxiety/panic and paranoia/suspiciousness. Poor ‘mindset’, poor ‘setting’ and mixing substances were most reported reasons for incidents. All but one respondent returned back to normality within 24 h.

**Conclusions:**

The results confirm psilocybin mushrooms are a relatively safe drug, with serious incidents rare and short lasting. Providing harm-reduction information likely plays a key role in preventing adverse effects. More research is needed to examine the detailed circumstances and predictors of adverse reactions including rarer physiological reactions.

## Introduction

Psilocybin-containing mushrooms (‘magic mushrooms’) have been used in some ancient cultures from prehistoric times (Carod-Artal, 2015), but more widespread use of the psychedelic did not start until the 1970s, following modern western research on psilocybin and increased knowledge about identification and cultivation of magic mushroom species (Andersson et al., 2009). The subjective effect of psilocybin is likely determined by partial agonism at the 5-HT2A receptor, which includes perceptual alterations (e.g. synaesthesia), increased emotional lability and changes in sense of self, time and space (Nichols, 2016; Vollenweider et al., 1998). Re-emerging experimental research on psilocybin in the past two decades has highlighted promise in the treatment of various mental health conditions and addictions (Rucker et al., 2018) as well as potential to increase well-being (Nicholas et al., 2018) and trait openness (MacLean et al., 2011) in healthy individuals.

Similar to other classical psychedelics – such as lysergic acid diethylamide( LSD; Holze et al., 2021; Kopra et al., 2022; Petranker et al., 2020) and ayahuasca (Lawn et al., 2017) – psilocybin is a physiologically safe substance relative to other psychoactive drugs with no evidence of neurophysiological deficits, organ damage or addiction potential (Johnson et al., 2018; Nichols, 2016). Acute physiological effects of psilocybin are mild. In normal doses, ranging from 3 to 30 mg of psilocybin corresponding to roughly 5–50 g of fresh mushrooms, it induces slight increases in breathing frequency, heart rate and blood pressure (Carbonaro et al., 2018; Gouzoulis-Mayfrank et al., 1999). Magic mushroom overdoses have additionally been associated with nausea, dizziness, shivering and abdominal pain (Van Amsterdam et al., 2011), though some of these symptoms are believed to be either psychosomatic or induced by phenylethylamine found in some species of mushrooms (Beck et al., 1998). Magic mushroom-related presentations to emergency departments do occur, but are usually rare and non-severe, dominated by mainly psychological symptoms with majority discharged after a short duration of stay (Leonard et al., 2018; Peden et al., 1981; Satora et al., 2005).

There are only three known deaths attributed to magic mushroom toxicity (Gerault and Picart, 1996; Lim et al., 2012). The estimated lethal dose of psilocybin is approximately 6 g of psilocybin drug substance, in essence 1000 times more than the threshold dose of 6 mg (Gable, 2004) and equivalent to about 10 kg of fresh mushrooms. Lethal overdose from eating mushrooms is, therefore, impractical as emesis would likely occur before absorption of toxic levels of the drug. However, variations in magic mushrooms’ potency between species, growing conditions and preservation can make estimating dosage difficult, hence increasing the risk of non-critical overdoses and challenging experiences.

The powerful psychological effects of psilocybin can, even in moderate doses, induce adverse reactions characterized by, for instance, anxiety, disorientation, fear, grief, paranoia and panic attacks (Barrett et al., 2016; Riley and Blackman, 2008; Van Amsterdam et al., 2011). Symptoms usually resolve within 6 h once the substance’s effects wear off, but a proportion report experiencing long-term detrimental effects on mental health (Carbonaro et al., 2016) and, in rarer cases, ‘flashbacks’, recurrences of perceptual alterations or other sensations experienced during the trip (Baggott et al., 2011; Carhart-Harris and Nutt, 2010). Furthermore, psilocybin-induced panic reactions and impairments in judgement and perception (Wittmann et al., 2007) can contribute to dangerous behaviour, accidents, self-harming and even suicidality (Carbonaro et al., 2016; Strassman, 1984). For instance, a small number of deaths from falls or jumps from tall buildings have been attributed to magic mushroom use (Van Amsterdam et al., 2011).

The promising research on psilocybin’s healing potential has given the substance positive visibility in the media in recent years. In experimental settings, with comprehensive participant screenings, carefully measured doses and supporting dosing environments, adverse reactions are indeed rare and outcomes generally positive (Rucker et al., 2018). Concerns have been raised that positive recovery stories might not only encourage psychedelic use but simultaneously overshadow information on safety precautions with potentially detrimental consequences (see the studies by Carhart-Harris et al., 2018; Yaden et al., 2021). An alarming example came from a recent report of a man with bipolar disorder who, inspired by reports of psychedelics’ therapeutic effects, injected homemade magic mushroom solution intravenously in an attempt to treat his depression, subsequently developing a multi-organ failure and spending 8 days in intensive care (Giancola et al., 2021).

To avoid human tragedies as well as their impact on psychedelics’ public image and progression of research, investigations on psychedelics’ risks are needed to guide public policy and harm-reduction initiatives. This study is an exploratory analysis of the occurrence, predictors and nature of adverse experiences resulting in emergency medical treatment (EMT) seeking following magic mushroom use, in a large international sample of Global Drug Survey (GDS) respondents. Specifically, we investigate the potential of demographic variables, mental health conditions, use patterns and previous magic mushroom experience as predictors of EMT incidents; and explore the symptom profile and recovery time from these experiences, concomitant use of other substances, perceived reasons for incidents and experiences’ impact on subsequent substance use.

## Methods

### Design

This investigation is one part of two articles looking at EMT seeking in response to psilocybin mushroom and LSD use in the same survey (Kopra et al., 2022). The reported methods are substantially similar within the two articles but are reproduced in each for the convenience of the reader.

The GDS is an annual, anonymous and encrypted online survey on substance use. It is advertised in social networking sites in collaboration with media partners and harm-reduction organizations. Using a self-nominating sampling method, the survey can effectively reach large amounts of respondents engaging in rarer practices and stigmatized behaviours, who would be difficult to access through representative sampling frames.

GDS 2017 was launched in November 2016 and was available until January 2017, in 10 languages. Participants were not remunerated. Full details about the survey design and recruitment, including related discussion on the survey’s utility can be found elsewhere (Barratt et al., 2017). Multi-institutional ethical approval was obtained from the King’s College London Research Ethics Committee (11671/001: GDS), University of Queensland (No. 2017001452) and The University of New South Wales (HREC HC17769) Research Ethics Committees. Access to the relevant sections of the GDS 2017 dataset (demographic data and sections on psychedelics) was obtained through a data sharing agreement with the GDS.

### Measures

At the start of the survey a wide range of demographical information was collected. In subsequent sections, participants were asked to indicate when they last used specific drugs from an extensive list of substances including magic mushrooms (never, in the last 30 days, between 31 days and 12 months ago, more than 12 months ago). Those indicating history of use with a drug were then redirected to sections with in-depth questions about the use of these substances. Among other questions, people who reported past year magic mushroom use were inquired about the number of days they used the drug in the last 12 months; whether they used magic mushrooms for the first time in the last 12 months; the number of magic mushrooms they normally take on a day of use; and whether they had sought EMT following the use of magic mushrooms in the past year. The number of EMT incidents experienced was not recorded.

Those indicating having sought EMT were then redirected to a further set of questions about that incident. Respondents were asked to tick the psychological and physiological symptoms they presented with from a list of 21, extrapolated from the available literature. Respondents were also asked about the number of magic mushrooms they had consumed during that session, what (if any) other substances they had taken, the duration of symptoms and whether they had required hospitalization. Participants were then asked about their perceptions of the reasons for the incident, picking a maximum of three out of six options; and asked about the impact of their experience on their use of magic mushrooms and other substances.

Towards the end of the survey, all participants were asked about their overall well-being and mental health, including whether they have ever been diagnosed with a mental illness. Ethical review boards required that participants were allowed to skip questions and leave empty responses if they did not want to complete specific items.

### Data analysis

Per-event risk of seeking EMT was calculated by dividing the number of participants indicating past year EMT seeking with the total number of times magic mushrooms was used among past year users, specifically

$$\frac{N\;\mathrm{participants}\;\mathrm{reporting}\;\mathrm{EMT}}{\left[\begin{array}{l} \mathrm{Mean}\;\mathrm{times}\;\mathrm{used}\;\mathrm{past}\;\mathrm{year}\\ \;\;\;\;\;\;\;\;\;\;\;\;\;\;\;\times N\;\mathrm{past}\;\mathrm{year}\;\mathrm{users} \end{array}\right]}$$

Only those participants responding to the EMT question were included when calculating the estimated total times used (the denominator), therefore, creating a representative sample of those proceeding and choosing to respond to the EMT question. While median and interquartile range (IQR) of past year magic mushroom uses were used for descriptive data, mean was used in the above calculation for the most accurate estimate of total times used in the sample.

Non-parametric statistics were utilized because dependent variables were found to be non-normally distributed. Mann–Whitney U tests were used to investigate whether there were differences in the age, past-year frequency of use or number of mushrooms commonly consumed on a day of use between EMT seekers and non-seekers. Pearson’s Chi-square (χ^2^) or Fisher’s exact tests were used to investigate associations between treatment-seeking status and gender (male/female), previous magic mushroom experience status (first time in the past year/experienced) and presence of mental health diagnosis (yes/no). Descriptive statistics and graphs were created to explore the experiences and symptom profiles of EMT seekers. In addition, two multiple correspondence analyses (MCA; see Supplementary Methods and Abdi and Valentin, 2007) were conducted to explore pattern of relationships between different self-reported symptoms and between different self-reported reasons for incidents.

For all statistical analyses, complete case analysis was used, that is, responses with missing data on the variables of interest were excluded from those analyses. Analyses were performed using SPSS IBM Statistics 26.

## Results

### Frequency and risk of EMT incidents

GDS 2017 received a total of 119,108 responses, of which 24.5% (*n* = 29,124) reported lifetime use of magic mushrooms; 43.0% (*n* = 12,534) of those who reported lifetime magic mushroom use reported having used magic mushrooms within the past year; demographic profile of these participants is presented in Table 1. Of the 9233 participants responding to the EMT question, 0.2% (*n* = 19) indicated they had sought EMT following magic mushroom use in the past year.

**Table 1.** Demographic profile of past year magic mushrooms users.

	*N*	Valid percentage
*N* (percentage of lifetime users)	12,534	43.0^a^
Age		
< 25	7890	62.9
25–34	3576	28.5
35+	1068	8.5
Gender		
Male	9866	78.7
Female	2668	21.3
Country of residence		
Germany	2591	20.7
United States	2134	17.0
Canada	1534	12.2
Denmark	1290	10.3
United Kingdom	1026	8.2
Other	3959	31.6
Ethnicity		
White	6729	88.6
Mixed	338	4.4
Hispanic/Latino	228	3.0
Other	303	4.0
Mental health diagnosis		
None	5636	73.1
Yes	2070	26.9
Depression^b^	1517	19.7
Anxiety^b^	1090	14.1
ADHD^b^	492	6.4
Bipolar^b^	202	2.6
Psychosis^b^	107	1.4
Other^b^	403	5.2
Use patterns		
Past-month users	2835	22.6
Past-year novel users	3543	38.9
Median days past-year use	2	1–3

ADHD: attention deficit hyperactivity disorder.

a Proportion of lifetime users.

b Those with a diagnosis were able to tick more than one diagnosis, hence the total number being larger than above row.

Among responders to the EMT question, mean number of times magic mushrooms were used in the past year was 3.72 (SD = 13.1), resulting in the estimated 34,347 number of total times used. With 19 EMT seekers, this gave the per-event risk estimate of 0.00055, indicating 0.06% or approximately 1 in 1800 of past-year magic mushroom intakes led to EMT seeking in this sample.

Due to the high number of past-year users who did not respond to the EMT question, the potential for attrition bias as well as the estimated prevalence of skipping the question was investigated by examining the subsample of past-year users who completed the whole survey. Among these 1895 past-year users, the response rate to the EMT question was 98% (*n* = 1857), demonstrating that a large majority of missing responses have likely occurred due to dropouts. Among these 1857 responders, 0.3% (*n* = 6) indicated having sought EMT, indicating a low chance of significant attrition bias when compared to the rate in the total sample (0.2%).

### Predictors of EMT seeking

Comparing the characteristics of EMT-seeking groups, Mann–Whitney U test revealed a significantly lower median age among EMT seekers (Median = 19, IQR: 18–23) compared to non-seekers (Median = 23, IQR: 20–27); Mann–Whitney test *z* = 3.09, *p* = 0.002. A Fisher’s Exact test showed no difference in the prevalence of EMT seeking between those with lifetime diagnoses of mental health conditions (0.2%) and those without (0.2%), *p* = 0.546. There was also no difference in the prevalence of EMT seeking between men (0.2%) and women (0.2%), *p* = 1.00.

Regarding patterns and history of use, Chi-square analysis showed no difference in the prevalence of EMT seeking between those who had used magic mushrooms for the first time the past year (0.2%) compared to those with previous experience (0.2%) χ^2^ (1, *N* = 9068) = 0.43, *p* = 0.512. There was also no significant difference between the number of commonly consumed mushrooms between seekers (Median = 4.0, IQR: 3.0–16.0) and non-seekers (Median = 4.0, IQR: 2.0–6.0), Mann–Whitney test *z* = 1.768, *p* = 0.077. There was also no significant difference in the past-year frequency of use of magic mushroom between EMT seekers (Median = 2.0, IQR: 1.0–6.5) and non-seekers (Median = 2.0, IQR: 1.0–3.0), Mann–Whitney test *z* = 1.479, *p* = 0.139.

### Symptom profile and nature of EMT incidents

**Figure 1.**
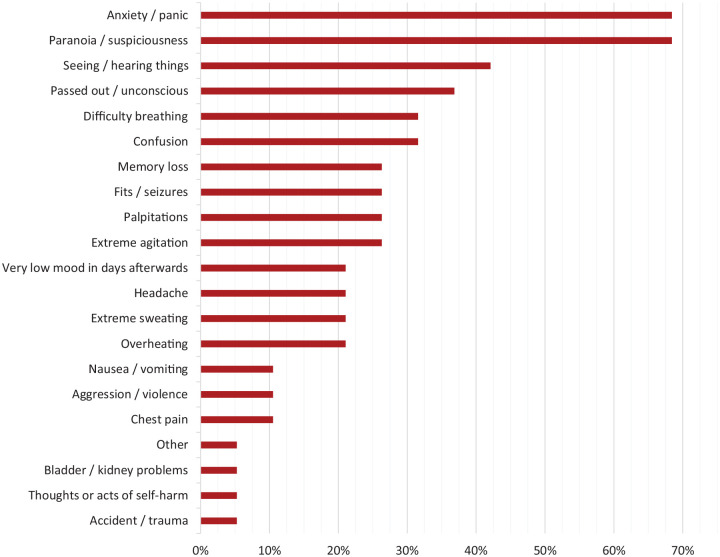
Self-reported symptoms.

Frequency of different reported symptoms are shown in Figure 1. The median (IQR) number of reported symptoms was 5.0 (2.0–8.0). The most commonly occurring symptoms were anxiety/panic (68%), paranoia/suspiciousness (68%), seeing/hearing things (42%) and passing out/unconscious (37%). Observation of the MCA factor map (Supplementary Figure S1) showed that frequently reported anxiety/panic and paranoia/suspiciousness were very closely related, as were seeing/hearing things and extreme agitation. Furthermore, palpitations, overheating, self-harm and difficulty breathing tended to co-occur. A fourth cluster identified showed an association between symptoms such as passing out, seizures, sweating, confusion, memory loss and very low mood afterwards.

Eight EMT seekers (42%; 95% CI: 20–64%) were admitted to hospital. Table 2 shows the length of time it took for respondents to feel back to normal; all but one respondent reported returning to normality within 24 h, and all respondents recovered within 7 days.

**Table 2.** Time to recovery.

	*N*	Valid percentage (95% CI)	Cu%
6 h	12	67 (46–88)	67
12 h	3	17 (0–34)	83
18 h	1	6 (0–17)	89
24 h	1	6 (0–17)	94
5–7 days	1	6 (0–17)	100

CI: confidence interval.

The median number of magic mushrooms consumed was 10.0 (IQR: 2.0–33.8). Table 3 shows other substances participants had used in the lead-up to the incident; 42% consumed no other substances, 37% of participants reported having used cannabis during the session while alcohol consumption was reported by 32%.

**Table 3.** Other substances used preceding the incident.

	*N*	Percentage
Cannabis	7	37
Alcohol	6	32
Cocaine	1	5
MDMA	1	5
Ketamine	1	5
Opioids	1	5
Nothing else	8	42

MDMA: 3,4-methylenedioxymethamphetamine.

Reasons for why participants thought the incident had happened are presented in Table 4. The most common reason was wrong mind-set (47%), followed by wrong place (37%) and mixing with other substances (37%). Observation of the MCA factor map (Supplementary Figure S2) indicated wrong mind-set and mixing substances were commonly reported together.

**Table 4.** Self-reported reasons for the incident.

	*N*	Percentage
Mood/mind-set	9	47
Mixed substances	7	37
Place/setting	7	37
Took too much	5	26
Don’t know	4	21
Not magic mushrooms	1	5

As a result of their experience, 58% of EMT seekers reported having cut down their magic mushroom use, while 37% reported no change in their magic mushroom use; 16% reduced and 0% increased their other illicit drug or alcohol use.

## Discussion

This article examined the prevalence and nature of adverse experiences leading to EMT seeking following the use of magic mushrooms, in a large global sample. Consistent with expectations, EMT seeking was very rare, occurring in only 0.2% of people reporting past-year use, with an estimated 1 in 1800 of magic mushroom ingestions leading to these incidents. Adverse experiences were short term with only one respondent experiencing effects lasting over 24 h. These results largely replicate previous literature supporting magic mushrooms’ safety and are reassuring considering both the wider public health perspective and the potential future medicinal use of psilocybin.

The most prevalent symptoms were psychological in nature, namely anxiety/panic and paranoia/suspiciousness. These are consistent with previous reports of the nature of adverse reactions to psilocybin and other psychedelics and have been discussed in depth elsewhere (Barrett et al., 2016; Van Amsterdam et al., 2011). However, a number of concerning physiological symptoms also occurred; passing out or going unconscious, difficulty breathing and seizures were reported by 37%, 32% and 26%, respectively. While difficulty breathing is commonly related to panic and anxiety, aetiology behind the two others is less clear. Rapid changes in blood pressure induced either directly by the drug or by psychological reactions, as well as dehydration or undernutrition are plausible triggers for losing consciousness under psilocybin; however, it is also plausible that some participants have merely had a transient memory loss or had fallen asleep during the experience. Passing out could theoretically also result from cardiac arrhythmias associated with prolonged QT interval induced by psilocybin, although high doses would be needed for this to occur (Dahmane et al., 2021). A number of seizures following magic mushroom consumption have been reported, the exact causes being largely unascertained (De Sagun and Tabunar, 2012; Leonard et al., 2018; McCawley et al., 1962). It is possible that pre-existing conditions, interactions with other substances or medications as well as consumption of poisonous mushrooms may have played a role in a proportion of such reactions; specifically, lithium has consistently been linked to severe adverse reactions to psychedelics including seizures and fugue states (Nayak et al., 2021). Regardless, we cannot confirm whether all reported seizures in the survey have been true epileptic seizures in contrast to pseudoseizures triggered by psychological factors.

Contribution of polysubstance use to adverse psychedelic experiences have been reported previously (Bienemann et al., 2020; Van Amsterdam et al., 2011). In both this study as well as in our investigation on LSD-related EMT experiences (Kopra et al., 2022), majority of respondents consumed other substances prior to seeking EMT, most commonly cannabis and alcohol. Although we do not know the overall prevalence of concurrent use of these substances among the whole magic mushroom user sample, and therefore cannot infer based on these statistics alone to what extent their use is specifically linked to adverse experiences in contrast to magic mushroom use per se (previously both cannabis and alcohol has been found to be frequently co-administered with psilocybin; Barrett et al., 2006; Kuc et al., 2021; Licht et al., 2012); over one-third of our respondents reported mixing substances as a reason for their adverse experience. In a previous survey study on psilocybin-related challenging experiences, 53% reported having used cannabis and 19% alcohol during or immediately before their experience (Carbonaro et al., 2016). Of note, Carbonaro and colleagues also found 26% of respondents in the survey used cannabis to attempt calming down; however, only half of these reported their attempts to be successful, and in optional open-ended textual responses several participants spontaneously reported cannabis having significantly exacerbated their difficult experience (Carbonaro et al., 2016). Cannabis can cause acute psychotic-like symptoms, also prevalent in this survey (D’Souza et al., 2004), further supporting cannabis may be more likely to exacerbate than alleviate magic mushroom-related adverse reactions. A recent prospective survey study suggested the association with cannabis and challenging psychedelic experiences to be dose-dependent, with low and medium doses of cannabis linked to less challenging experiences, and high doses with more challenging experiences (Kuc et al., 2021).

Besides mixing substances, being in the wrong mood/mind-set and place/setting were among the most commonly reported reasons for incidents, consistent with extensive literature on the importance of these factors for preventing adverse reactions to psychedelics (Carhart-Harris et al., 2018). However, a significant proportion indicated uncertainty regarding the reason of the incident, two times higher than in our investigation on LSD (21% vs 10%; Kopra et al., 2022). Adverse reactions to psychedelics can occur even in optimized settings with adequate preparation (Carbonaro et al., 2016; Haijen et al., 2018). Anecdotal reports have described magic mushrooms’ effects as less predictable and less sensitive to ‘set and setting’ compared to LSD (Grey and Aremu, 2020; Third Wave, 2015). Therefore, despite being less likely to cause serious adverse experiences (Kopra et al., 2022; Leonard et al., 2018), higher proportion of these might be unexpected and triggered by unknown factors. More evidence is, however, needed to confirm the hypothesis. Results from the first experimental studies comparing the effects of LSD and psilocybin head-to-head are yet to be published (NCT03604744; NCT04227756).

The only predictor of EMT incidents in this study was younger age. Previous studies on psilocybin had similarly shown younger age to predict more challenging experiences (Carbonaro et al., 2016; Studerus et al., 2012). The association was also found in our investigation on LSD-related EMT incidents (Kopra et al., 2022), where we suggested potential explanations for the association including lower risk-averseness and higher impulsivity that could link to more risky drug use behaviours (Spear, 2000; Steinberg et al., 2008), as well as relative difficulty of emotional regulation in some younger people (Carstensen et al., 2011; Williams et al., 2006). Previous experience with psychedelics did not predict risk of incidents in either of our two investigations. Carbonaro et al. (2016) had previously found a negative correlation between past hallucinogen use and difficulty of psilocybin-induced challenging experiences; however, although significant, this association was small in magnitude. People with more experience with psychedelics do not, therefore, appear to be protected from adverse experiences but should remain mindful of the risks brought by experimenting with challenging environments, increasing dosages and mixing substances.

There was no indication for a higher risk of EMT seeking in people with lifetime mental health diagnoses. Previous research has suggested associations between serious adverse reactions to psychedelics and presence of mental health conditions; however, it is possible the risk is less pronounced in common mental health conditions compared to psychotic or bipolar disorders (Cohen, 1960; Strassman, 1984). Psychedelics, specifically psilocybin, show early promise in the treatment of depression, anxiety and addictions, highlighting the relationship between mental health and the nature and outcomes of psychedelic experiences is highly multifaceted, affected by various contextual factors and traits beyond the presence of psychopathology (Aday et al., 2021; Carhart-Harris et al., 2018). People who use psychedelics are a self-selective group and some individuals with certain predispositions may instinctively know not to take psychedelics or to use them with more care, therefore, making it more difficult to find predictors of effects from naturalistic use. It is also plausible that some of those with lifetime diagnoses in our survey were recovered or in remission during the reporting period. Regardless, the present findings conflict with our investigation on LSD-related EMT presentations, where mental health conditions did predict EMT incidents with a large effect size (Kopra et al., 2022). Given the low number of magic mushroom incidents, the present finding could have been a false negative; alternatively, it is not ruled out that differences between susceptibility to adverse LSD and psilocybin experiences exist, an area which would require further investigation.

The low rate of emergency presentations is in line with both expert analyses and assessments of people using substances, rating psilocybin as the drug of lowest harm among commonly used recreational substances (Carhart-Harris and Nutt, 2013; Nutt et al., 2010; Van Amsterdam et al., 2010). The prevalence of EMT seeking in this study was approximately five times lower when compared to LSD-related EMT incidents in the same survey (Kopra et al., 2022). Similarly, an analysis of LSD and magic mushroom exposures reported to United States poison centres observed lower occurrence of major incidents and hospital admissions associated with the latter (Leonard et al., 2018). Potential explanations for these differences include higher potency of LSD (Isbell, 1959) that likely increases the risk of accidental overdoses, whereas extreme overdoses from mushroom consumption is practically very difficult; ‘taking too much’ was, indeed, a less commonly reported reason of EMT incidents in this study compared to our report on LSD (26% vs 40%; Kopra et al., 2022). In addition, psilocybin’s duration of effects is two times shorter compared to LSD (Nichols, 2016), decreasing the risk of prolonged adverse experiences. Other differences in the substances’ pharmacology and subjective effects have been reported (Giacomelli et al., 1998; McCartney et al., 2021), but further, experimental research is needed to confirm these and how they may contribute to the substance’s differential safety profile.

While the low incidence of EMT incidents is a positive finding it can also be regarded as a limitation in the study, as predictors of incidents were difficult to establish and nature of experiences could only be analysed from 19 participants. Specifically, higher using frequency and higher dose showed a trend towards increasing the risk of incidents; however, very large samples would be needed for enough power to detect these and potential other predictors. Continued investigation on less severe (and more common) adverse experiences can contribute to our knowledge about serious reactions which, based on reported symptoms, are often similar in nature but only more intense. Investigation is, however, also required on the aetiology of some more rare emergencies including seizures; reaching people with such experiences for more thorough qualitative assessments could provide insights on their causes and impact, and supplement data from official records and large quantitative surveys.

It is, regardless, reassuring that despite varying symptomology, all but one respondent reported being back to normality within 24 h. However, we cannot confirm whether ‘back to normality’ has, for some, meant only the resolution of acute drug effects and complications, and not necessarily the absence of longer lasting psychological impact. Although most people reporting challenging psychedelic experiences also cite resulting therapeutic value and benefits to their well-being (Carbonaro et al., 2016), they can also be traumatizing and lead to psychological distress especially when negative aspects dominate the trip and where there is no adequate support during and after the experience (Carbonaro et al., 2016; Gashi et al., 2021; Gorman et al., 2021). Training mental health professionals in psychedelic integration and reducing stigma and criminalization associated with psychedelic drugs is important for encouraging people to come forward and seek and receive help when this is needed (Gorman et al., 2021).

Several other limitations need to be considered when appraising this study. Self-nominating, non-probability sampling is subject to sampling and volunteer biases that reduce sample representativeness. In essence, inherent differences may exist between people who are reached by the recruitment and choose to volunteer to participate compared to those who are not. Differences could also occur among those who drop out early or who choose not to respond to specific questions. Although our subanalysis among survey completers indicated a low chance of significant attrition bias, we cannot ascertain whether the rare case of skipping the EMT question may have been disproportionately more common among actual EMT seekers or non-seekers, therefore, biasing the rate of EMT seeking to either direction. Furthermore, retrospective self-reports are often affected by recall and response biases; answers might be influenced by, for instance, substances’ effects on cognition or by personal opinions about drugs. In addition, the options for perceived reasons for the incident did not include ‘Other’ nor a possibility for an open-ended text response; therefore, the question and the limited options may be leading the respondents’ answers. Limitations concerning sampling, participation bias and response bias are discussed in more depth in our twin articles (Kopra et al., 2022); see also study by Barratt et al. (2017).

Our survey cannot confirm the purity or potency of magic mushrooms and potential other substances used. Even if correct substances have been reported, contribution of each in inducing the symptoms cannot be ascertained; similarly, we cannot confirm the extent to which psilocybin versus other compounds in magic mushrooms, such as phenylethylamine, have contributed to the experience – although the purpose of the article is to investigate naturalistic magic mushroom use and not the effects of pure psilocybin. Furthermore, our variable ‘number of mushrooms’ is a vague indicator of quantity used; besides the high variation in sizes of mushrooms, many people who use mushrooms record their use in grams or consume readily grinded, dried mushrooms and are, therefore, not aware of the number of mushrooms they have used. Finally, our survey could not establish the exact circumstances surrounding the incidents or the determining factors leading to EMT seeking in each case.

Regardless of limitations, this investigation has provided valuable insights on the occurrence and nature of magic mushroom–related serious adverse experiences, from the world’s largest survey on drug use. Magic mushrooms are relatively innocuous substances and rarely cause harm to the individual consuming them nor to other people. Most adverse reactions are short-lived, and their risk can be minimized with certain safety precautions. The results are reassuring from the public health perspective, and support the reassessment of psilocybin’s legal status to aid the delivery of clinical research and effective harm-reduction services.

## Supplemental Material

sj-docx-1-jop-10.1177_02698811221084063 – Supplemental material for Adverse experiences resulting in emergency medical treatment seeking following the use of magic mushrooms Click here for additional data file. Supplemental material, sj-docx-1-jop-10.1177_02698811221084063 for Adverse experiences resulting in emergency medical treatment seeking following the use of magic mushrooms by Emma I Kopra, Jason A Ferris, Adam R Winstock, Allan H Young and James J Rucker in Journal of Psychopharmacology
