# Supplementary material for: Adverse experiences resulting in emergency medical treatment seeking following the use of magic mushrooms
Source: J Psychopharmacol. 2022 Apr 7;36(8):965–73. doi: 10.1177/02698811221084063 (PMC9353971; doi:10.1177/02698811221084063)
Supplement: sj-docx-1-jop-10.1177_02698811221084063 – Supplemental material for Adverse experiences resulting in emergency medical treatment seeking following the use of magic mushrooms [file sj-docx-1-jop-10.1177_02698811221084063.docx]

**Supplementary Methods – Multiple Correspondence Analysis**

Multiple Correspondence Analysis (MCA) is an exploratory data analysis tool used to identify patterns and associations between multiple categorical variables. It is a form of dimension reduction method and often seen analogous to Principal Component Analysis (PCA) for quantitative data. As in PCA and Factor Analysis, the first dimension explains most variance in the dataset with each additional dimension explaining less. Most commonly 2- or 3-dimensional solution is used, as each added dimension becomes less important and complicates interpretation of the data.

MCA produces a scatter plot or a factor map to visualize relationships between variables. Most correlated variables – in essence, variables or options that have commonly been selected together – occur near each other; while uncorrelated variables are plotted far from each other. The most common responses are generally closest to the centre of the grid or the origin (0:0) and conversely less common responses are further away. Clouds are drawn over the factor map to highlight clusters of highly correlated variables.


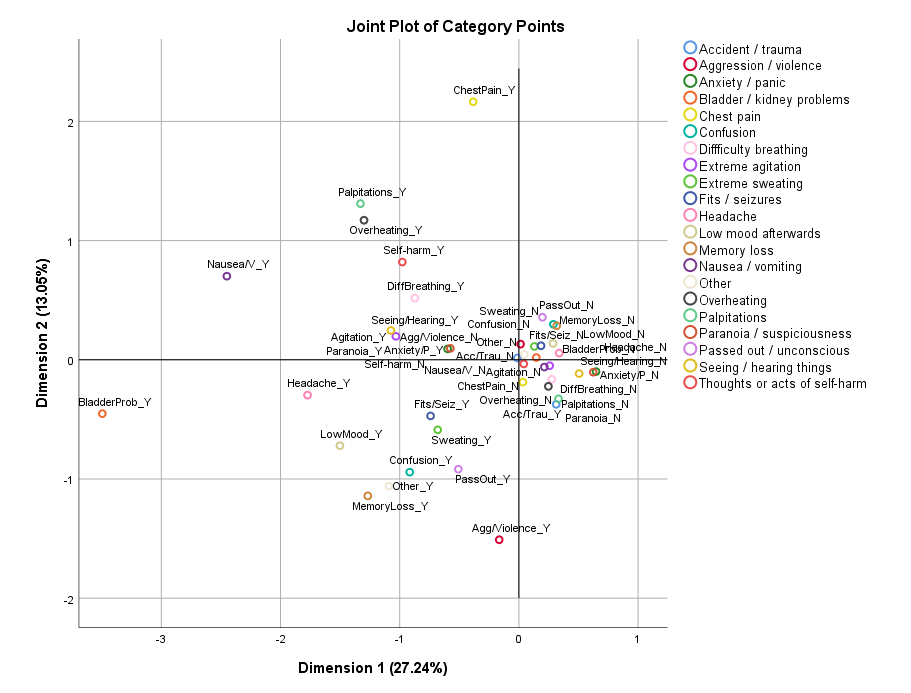
**Supplementary Figure S1. MCA Factor Map of Reported Symptoms**

3

2

4

1

A two-dimensional model explained a total of 40.3% of the variance (27.24% + 13.05%). “Yes” responses (indicated with a “Y”) are generally clustered in the left side of the y axis, and “No” responses (“N”) to the right. “Yes” responses are more spread out, reflecting that presenting a symptom was generally less common than not presenting one, as demonstrated by the median number of symptoms (5 vs. total options 21). At the top left quadrant, a cluster formed of palpitations, overheating, self-harm and difficulty breathing was identified (Cloud 1). Below this, two small clouds show strong associations between seeing/hearing things and extreme agitation (Cloud 2); and commonly reported psychological symptoms anxiety/panic and paranoia/suspiciousness (Cloud 3). The bottom left quadrant shows a relationship between symptoms such as passing out, seizures, sweating, confusion, memory loss and very low mood afterwards (Cloud 4).

**Supplementary Figure S2. MCA Factor Map of Reasons for Incidents**


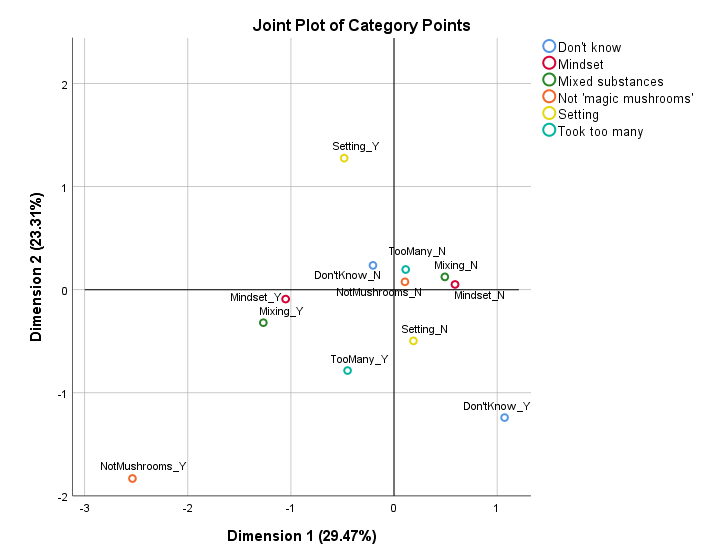


1

A two-dimensional model explained a total of 52.8% of the variance (29.47% + 23.31%). “Yes” responses generally clustered to the left side of y-axis; except for “Don’t’ know” on the bottom right corner, demonstrating selecting this option was expectedly linked to not ticking other reasons. At the bottom left quadrant, Cloud 1 shows mixing substances and wrong mindset commonly co-occurred.
